# Epidemiology of *Salmonella enterica* subspecies *enterica* serotypes, isolated from imported, farmed and feral poultry in the Cayman Islands

**DOI:** 10.3389/fvets.2024.1331916

**Published:** 2024-02-09

**Authors:** Simon Watler, Felix N. Toka, Hélène Lardé, Antoinette Johnson, Patrick Butaye

**Affiliations:** ^1^Department of Environmental Health, Ministry of Health and Wellness, Grand Cayman, Cayman Islands; ^2^Department of Biomedical Sciences, Ross University School of Veterinary Medicine, Basseterre, Saint Kitts and Nevis; ^3^Department of Clinical Sciences, Ross University School of Veterinary Medicine, Basseterre, Saint Kitts and Nevis; ^4^Faculty of Veterinary Medicine, Department of Pathobiology, Pharmacology and Zoological Medicine, Ghent University, Merelbeke, Belgium; ^5^Department of Infectious Diseases and Public Health, Jockey Club College of Veterinary Medicine and Life Sciences, City University of Hong Kong, Kowloon, Hong Kong SAR, China

**Keywords:** *Salmonella enterica*, poultry, Cayman Islands, whole genome sequencing, public health, non-typhoidal salmonella

## Abstract

Non-typhoidal *Salmonellae (NTS)* are common foodborne pathogens throughout the world causing acute gastroenteritis. Compared to North America and Europe, there is little information on NTS in the Caribbean. Here we investigated the prevalence and characteristics of NTS present in the local poultry of the Cayman Islands to determine the public health risk. In total, we collected 156 samples. These were made up of boot swabs of 31 broiler farms and 31 layer farms (62 samples), paper bedding from 45 imported chick boxes, and 49 pooled cecum samples from feral chickens, each sample representing 10 individual chickens. *Salmonella* was isolated using the ISO 6579 protocol and isolates were characterized using Whole Genome Sequencing (WGS) analysis. Eighteen *Salmonella* isolates were obtained and comprised six *S. enterica* subspecies *enterica* serotypes and one subspecies *houtenae* serotype. Serotypes were: *S.* Kentucky (*n* = 9), *S.* Saintpaul (*n* = 5), *S.* Javiana (*n* = 1), *S.* Senftenberg (*n* = 1), *S.* Poona (*n* = 1) and *S.* Agona (*n* = 1). *S*. Kentucky strains were all ST152 and clonally related to poultry strains from the United states. *S*. Saintpaul ST50 strains showed clonality to North American strains. Over half of the strains (*n* = 11) contained resistance genes to at least two antibiotic groups and five strains were MDR, mainly those from imported day-old chicks. The *bla*_CMY-2_ gene was found in *S.* Kentucky from day-old chicks. Strains from feral poultry had no acquired AMR genes. While serotypes from feral poultry have been identified in human infections, they pose minimal risk due to their low virulence.

## 1 Introduction

Non-typhoidal *Salmonellae* (NTS) cause over 93 million infections worldwide, with approximately 80.3 million attributed to food-borne transmission (1). The economic burden of salmonellosis in the United States alone has been estimated at $3.7 billion US dollars per year, ranking 1st among 15 other foodborne pathogens (2). NTS infections are one of the leading causes of death and hospitalizations amongst foodborne infections in the U.S (3). Symptoms are usually mild with fever, abdominal pain and diarrhea, but can becoming life-threating mainly in children under five years of age, immunocompromised and elderly people (4, 5). NTS infections usually do not require treatment, are self-limiting and patients can make a full recovery.

Human health is one of the three major components of the One Health concept, along with animal health and environmental health (6). The One Health concept emphasizes the close relationship between animal and human health as well as the environment and its ecosystems, to ensure optimal and sustainable balance between these three components. This is achieved by a collaborative, multi-discipline effort of different sectors to effectively address global health problems and create policies for long-term sustainability (7).

One critical threat that undermines the One Health concept is antimicrobial resistance (AMR), in which antimicrobials become ineffective, increased complications in treatment and the rise of ‘superbug’ microbes (8, 9). In 2019, AMR was declared among the top 10 threats to global health according to the World Health Organization. AMR is of great concern among NTS since it increases treatment difficulties. However, there is significant variation in the prevalence of AMR, depending on geographical location, time, and serotype (10). Of grave concern is the resistance against third-generation cephalosporins and fluoroquinolones (According to the World Health Organization’s classification, these are deemed as Highest Priority Critically Important Antimicrobials), which are currently the preferred antibiotic treatment of severe human NTS infections in the both the United States and EU (11). In the USA there is a steady decline of AMR in NTS. In 2019, 78% of *Salmonella* isolated from clinical cases did not exhibit any antibiotic resistance (12), in comparison to previous years of 70% in 2014 and 76% in 2015 (13, 14). While ciprofloxacin resistance in the United States has remained steadily under 0.5% since 2018, decreased susceptibility to ciprofloxacin has been steadily rising starting from 0.2% of isolates in 1996 to 10.7% of isolates reported in 2019 (12). Resistance against ceftriaxone remained stable at 3% in 2019 (15), while in 2009, 20.8% of the *Salmonella* isolated from chickens were resistant to third generation cephalosporins. This contrasts to human clinical infections reported during those years, with ceftriaxone resistance consistently around 2.5% for the last decade (16).

Of the approximately 2,500 serotypes of *Salmonella*, only a small number of serotypes accounts for 99% of human and animal clinical cases. Common serotypes associated to human salmonellosis change over time, vary by geographical distribution and its ability to affect hosts (17). Currently, the most common serotypes implicated in human disease include *S. typhimurium* and *S. enteritidis* worldwide (18).

NTS are part of the commensal flora of the intestinal tract of cold-blooded animals such reptiles, turtles and amphibians (19) as well as some birds (20). These animals may infect domesticated animals when there are no rigorous biosecurity measures.

The Caribbean Public Health Agency (CARPHA), the major public health agency within the Caribbean comprised of 26 Caribbean nations, reported an average of 564 annual clinical cases of NTS infections during the years 2005–2012, but there has been a downward trend since 2010 (21). Between the years 2005–2012, a total of 146 different *Salmonella* serotypes were identified as causes of infections, with half of the infections attributed to *S. enteritidis* and *S. typhimurium* (21, 22).

NTS have been shown to be present in Caribbean wildlife including iguanas (23, 24), mongooses (25, 26), cane toads (27), crabs (28), sea turtles (29–31), snakes (32), albeit at low prevalence. Serotypes present varied between cold-blooded and warm-blooded animals. In farmed animals, the focus was mainly on poultry, and showed a prevalence rate range between 6.5–26.7% (33) and the prevalence in pigs in Surinam was 9% (34). Majority of poultry studies are reported from Trinidad and Tobago (35–40). In the Cayman Islands 10 different serotypes were found in native iguana and *S. enterica* serotype Saintpaul was the most frequently isolated serotype (23). In the Caribbean, biosecurity is not optimal as most animal houses are open houses allowing the entry of some of wildlife. Given that the majority of human infections often stem from poultry and eggs, the primary objective of this study was to assess the potential public health risk associated with salmonellosis in humans in the Cayman Islands, due to poultry.

Since most chickens in the Cayman Islands originate from imported embryonated eggs, we investigated whether *Salmonella* could be introduced in the local poultry by import. In addition, we looked whether feral chickens could be a source of salmonellosis for farmed poultry.

## 2 Methodology

### 2.1 Sampling

**Figure 1:**
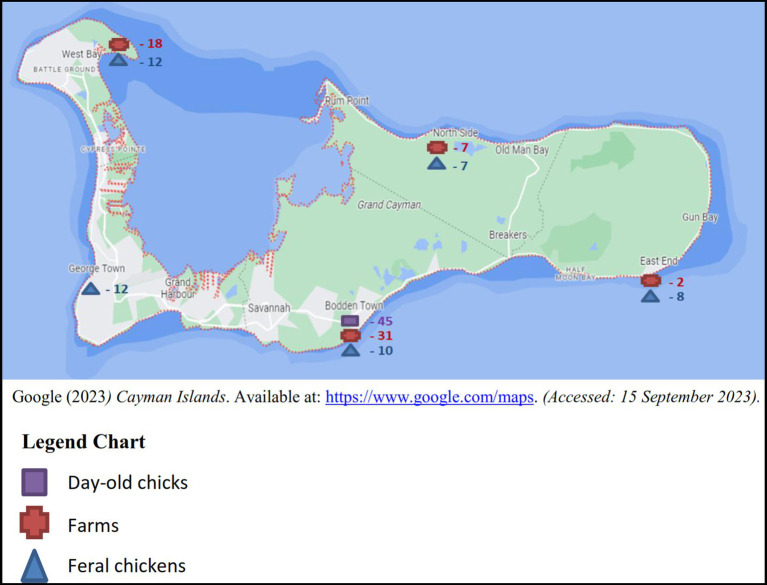
Number of samples of each sample type from the five districts of Grand Cayman.

**Figure 2:**
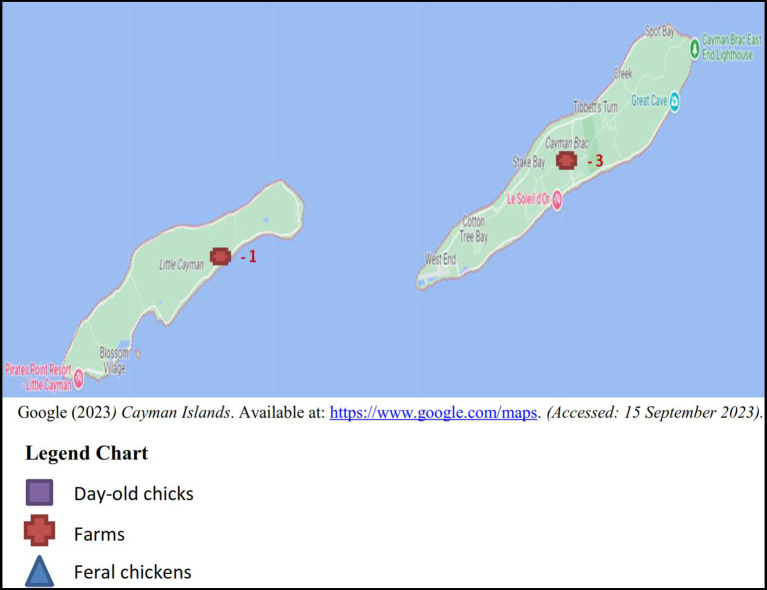
Number of samples of each sample type from Cayman Brac and Little Cayman.

Between the years 2017 and 2018, sampling was conducted on a monthly basis totaling a minimum of four boot swabs, four pooled samples of 10 feral chickens per month and batches of imported day-old chicks as received. The samples originated from the five districts in Grand Cayman: West Bay, George Town, Bodden Town, East End and North Side (Figure 1), 3 samples (2 layer and 1 broiler farm) were taken from Cayman Brac and a single sample from Little Cayman (Figure 2).

For each monthly sampling event, a minimum of two pairs of boot swabs were sampled at each farm (broiler or layer), with a maximum of four boot swabs per farm depending on its size. Feral chickens of each district were captured, humanely euthanized and cecum contents were pooled from 10 feral chickens representing one sample. Lastly, paper bedding material from each chick-box of day-old chicks was sampled prior to distribution to farmers.

31 boot swab samples were taken in according to the Commission Regulation (EU) No. 517/2011 (41), from both broiler and layer farms. Since the population size of feral chickens in the Cayman Islands is undetermined, we collected 49 pooled cecum samples, each representing 10 feral chickens. 45 paper bedding samples composed from 6 different shipments in 2018 were taken from the boxes in which the day-old chickens were imported.

*Salmonella* was isolated using ISO Standard 6,579 annex D (42). Briefly, a 1/10 (W/V) suspension of 25 g paper bedding, cecum content, or boot swabs were mixed with 225 mL Buffered Peptone Water (BD Life Sciences, Fanklin Lake, NJ, United States). After stomaching, they were aseptically incubated at 37 ± 1°C for 18 ± 2 h, then 0.1 mL was added to 10 mL Rappaport Vassiladis R10 (RV) broth (BD Life Sciences, Fanklin Lake, NJ, United States) and incubated at 41.5°C for 24 ± 4 h. One ml of resuscitation broth was added to 10 mL Tetrathionate broth (TB) base (Fisher Scientific, Waltham, MA, United States) and incubated at 37°C for 24 ± 4 h. Thereafter, 10 μL of enrichment broth was streaked on Xylose Lysine Deoxycholate (Fisher Scientific™, Waltham, MA, United States) and Brilliant Green Agar (Fisher Scientific™, Waltham, MA, USA) and incubated at 37 ± 1°C for 24 ± 4 h. Presumptive *Salmonella* colonies were inoculated on Blood Agar (Fisher Scientific™, Waltham, MA, United States) at 35 ± 2°C for 18 to 72 h. Suspected colonies were identified using API 20E test strips (bioMérieux, Marcy l’Étoile, France), which were incubated at 36 ± 2°C for 18–24 h and the *Salmonella* Express Plate (3M™ Petrifilm™, Maplewood, Minnesota, United States), incubated at 41.5°C for 24 ± 4 h. Lastly, *Salmonella* agglutination tests were conducted to determine serogroup using the Wellcolex Color Salmonella Rapid Latex Agglutination Test Kit (Thermo Fisher Scientific, Waltham, MA, USA). Serogrouped strains were selected for WGS.

### 2.2 Whole genome sequencing and sequence analysis

Purified strains were sent to Macrogen (Seoul, South Korea) for DNA extraction and sequencing on an Illumina platform using a TruSeq Babi DNA kit, 151 bp long paired end sequencing. Sequences were submitted to NCBI under bio-project PRJNA765319 with accession numbers: SAMN21553720 and SAMN22875821 - SAMN22875840. Quality control has been performed on the raw data using Phred and on the assembled data using Quast.

Illumina sequences were trimmed and assembled using SKESA (43) and annotated with PROKKA (44). *Salmonella* serotypes were identified using Seroseq (45) and SISTR (46). The Multi-Locus Sequence Typing (MLST) profile was determined using ‘mlst 2.0’ (47). Phylogeny was determined with strains from around the world using The Phylogenetic Tree Building Service on the PATRIC server (currently Bacterial and Viral Bioinformatics Resource Center) (48) *last accessed November 10th, 2022* and base differences between strains were quantified by CSIPhylogeny (49). The accessory genome was analysed using Resfinder (50) to identify antimicrobial resistance genes and associated antibiotic resistance of the strains. Multi-drug resistance (MDR) was defined as strains showing resistance to three or more different classes of antimicrobials (51). Plasmids were identified with PlasmidFinder 4.0 (52). PHASTER (53, 54) was used to identify prophage proteins present in bacterial sequences. Virulence genes were determined by Virulence Factor Database (VFDB), (55) and SPIFinder (56).

### 2.3 Statistical analysis

Confidence intervals to measure the prevalence of *Salmonella* in poultry, in the Cayman Islands were calculated using exact binomials in an Excel file. Differences between groups were determined using the chi square test.

## 3 Results

### 3.1 Prevalence of salmonella in poultry in the Cayman Islands

Out of 156 samples, 18 were found to be positive for *Salmonella,* resulting in an overall prevalence rate of 11.5% (confidence interval 11.2–11.8%) (Table 1). Each positive originating from a farm source, broilers or layers, were isolated from separate farms, resulting in four positives from four individual farms. With four farms positive for *Salmonella* of the 27 total farms sampled, the prevalence rate is 14.8% (confidence interval 13.8–15.8%). Six of the 49 pooled cecal samples of feral chickens were positive and eight of the 45 paper bedding samples of imported day-old chicks were positive. Broiler farms showed the lowest prevalence of 3.2% (confidence interval 3.1–3.3%) whereas imported day-old chicks showed the highest prevalence of 17.8% (confidence interval 16.8–18.8%).

**Table 1:** Number of samples collected, number positive for *Salmonella* and corresponding prevalence with confidence intervals.

Origin	N* of samples	N* of positives	Prevalence	Confidence intervals
Broiler	31	1	3.2%	3.1–3.3%
Layer	31	3	9.7%	9.2–10.2%
Day-old chicks	45	8	17.8%	16.8–18.8%
Feral	49	6	12.2%	11.7–12.8%
Total	156	18	11.5%	11.2–11.8%

*N-Number.

### 3.2 Serotyping and MLST

**Figure 3:**
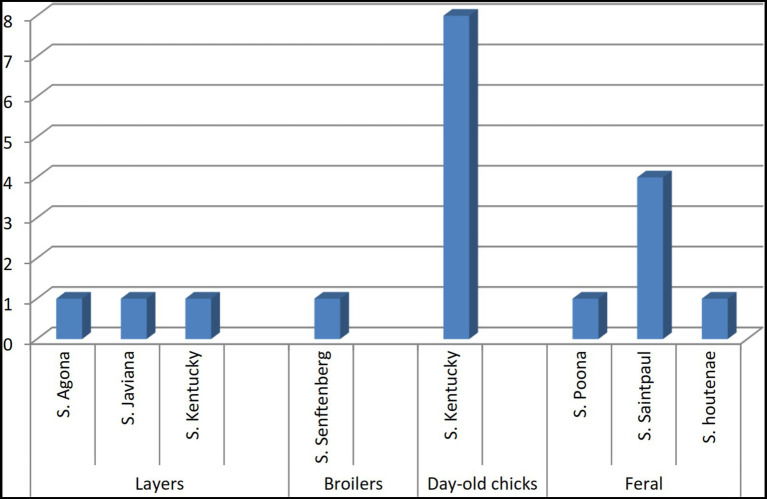
Distribution of *Salmonella* serotypes isolated from imported, farmed, and feral poultry, represented by sample origin.

**Figure 4:**
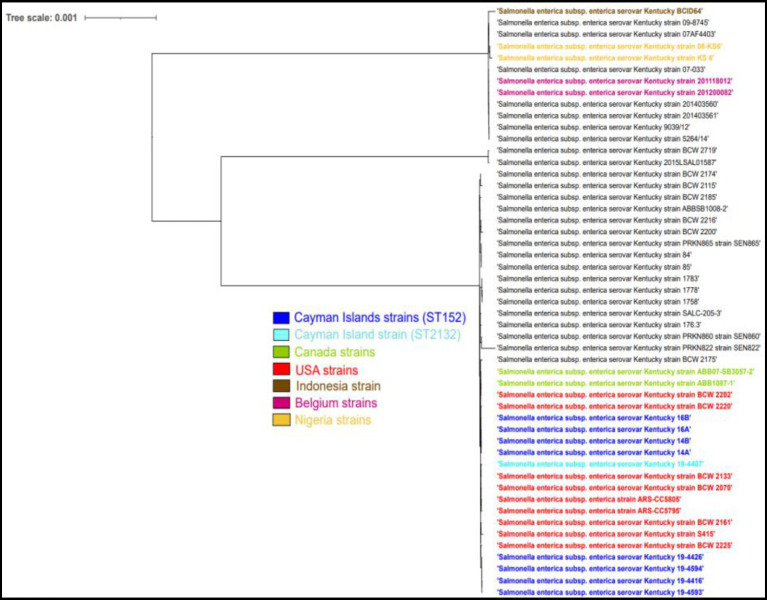
Phylogenetic tree analysis of Cayman Islands ST152 (dark blue) and ST2132 (light blue) strains and other worldwide ST152 strains (black) of *S*. Kentucky.

We identified seven different *Salmonella* serovars, with *S.* Kentucky being the most frequently isolated. *Salmonella* found in paper bedding/transport material from day-old chicks was exclusively *S.* Kentucky (Figure 3). Additionally, a *S.* Kentucky strain was isolated from a layer farm but none was found in feral poultry, where *S.* Saintpaul was the predominant serotype (Figure 4). Of the 9 *S*. Kentucky strains, eight were sequence type ST152 and one strain was ST2132, which is one allele (*purE*) different from ST152. *S*. Saintpaul strains (*n* = 4) were all sequence type ST50. The remaining strains (*n* = 5) were different *Salmonella* serotypes (Table 2).

**Table 2:** Characteristics and sample type from which the *Salmonella* strains were isolated.

Sample ID	District	Origin	Serotype	MLST 2.0
14A	Bodden Town	Imported Day Old Chicks	Kentucky	ST 152
14B	Bodden Town	Imported Day Old Chicks	Kentucky	ST 152
16A	Bodden Town	Imported Day Old Chicks	Kentucky	ST 152
16B	Bodden Town	Imported Day Old Chicks	Kentucky	ST 152
19–4,407	Bodden Town	Imported Day Old Chicks	Kentucky	ST 2132
19–4,416	Bodden Town	Imported Day Old Chicks	Kentucky	ST 152
19–4,426	Bodden Town	Imported Day Old Chicks	Kentucky	ST 152
19–4,593	Bodden Town	Imported Day Old Chicks	Kentucky	ST 152
19–4,392	Bodden Town	Broiler	Senftenberg	ST 14
S25	Bodden Town	Layer	Agona	ST 13
19–4,409	North Side	Layer	Javiana	ST 371
19–4,594	Bodden Town	Layer	Kentucky	ST 152
19–4,384	Bodden Town	Feral	Saintpaul	ST 50
19–4,397	George Town	Feral	Saintpaul	ST 50
19–4,417	West Bay	Feral	Poona	ST 447
19–4,411	East End	Feral	Saintpaul	ST 50
19–4,412	George Town	Feral	Saintpaul	ST 50
19–4,422	George Town	Feral	Subspecies *houtenae*	ST 162

### 3.3 Phylogenetic analysis

**Figure 5:**
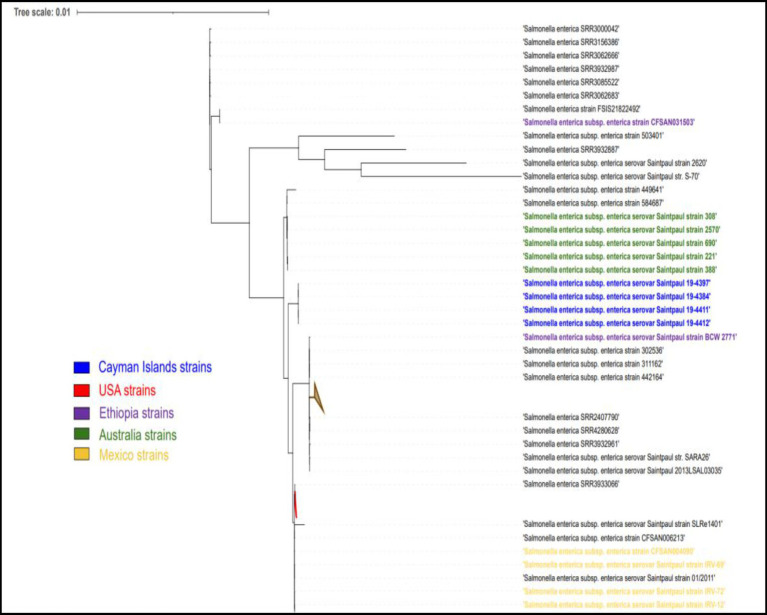
Phylogenetic tree analysis of Cayman Islands ST50 strains (blue), and other worldwide ST50 strains (black) of *S*. Saintpaul.

The single nucleotide polymorphism (SNP) analysis of the *S.* Kentucky strains, taken from imported day-old chicks and the layer farm, showed a variation ranging between 0 and 112 SNPs. When compared to ST152 *S*. Kentucky strains from around the world, the strains clustered closely with strains from North America (Figure 4). The S. Saintpaul strains showed less variation with 16–22 SNP differences, however, they formed a separate cluster from other ST50 strains from around the world (Figure 5).

### 3.4 Antimicrobial resistance genes

All 18 strains contained at least the *aac(6′)-Iaa* gene known to be present in the core genome of all *Salmonella* strains, giving resistance to amikacin and tobramycin (Table 3). Seven strains did not contain any other resistance genes. Only the serotypes *S.* Kentucky and *S*. Senftenberg contained more than one aminoglycoside resistance gene. Eleven strains showed additional resistance genes apart from those for aminoglycosides. All but one of these 11 strains were resistant to tetracyclines, mediated by the *tet*(B) gene and in one by the *tet*(A) gene. One strain was found to carry the *sul1* gene, encoding resistance to sulfonamide antibiotics, another was found to have chloramphenicol resistance gene, *catA3* and another strain to have fosfomycin resistance via *fosA7_1* gene. Lastly, two genes encoding resistance to β-lactam antibiotics were present: *bla*_TEM-1B_ in the single *S.* Senftenberg and *bla*_CMY-2_ in 4 *S.* Kentucky strains from one-day old chicks. Four *S.* Kentucky strains and one *S*. Senftenberg strain were multi-drug resistant.

**Table 3:** AMR genes and plasmid replicons detected in the *Salmonella* strains.

Sample #	Serotype	Antimicrobial resistance genes					Plasmids
		Amino-glyocide	Tetra-cycline	Sulfon-amide	Beta-Lactam	Fosfo-mycin	Plasmid replicons
14A	Kentucky	*aph(6)-Id, aph(3″)-Ib, aac(6′)-Iaa*	*tet*(B)				ColpVC, IncFIB, IncFII, IncX1
14B	Kentucky	*aph(6)-Id, aph(3″)-Ib, aac(6′)-Iaa*	*tet*(B)				ColpVC, IncFIB, IncFII, IncX1
16A	Kentucky	*aph(6)-Id, aph(3″)-Ib, aac(6′)-Iaa*	*tet*(B)				ColpVC, IncFIB, IncFII, IncX1
16B	Kentucky	*aph(6)-Id, aph(3″)-Ib, aac(6′)-Iaa*	*tet*(B)				IncFIB, IncFII, IncX1
19–4,407	Kentucky	*aph(6)-Id, aph(3″)-Ib, aac(6′)-Iaa*	*tet*(B)				IncFIB, IncFII, IncX1
19–4,416	Kentucky	*aph(6)-Id, aph(3″)-Ib, aac(6′)-Iaa*	*tet*(B)		*blaCMY-2*		IncFIB, IncFII, IncI1-I(Alpha), IncX1
19–4,426	Kentucky	*aph(6)-Id, aph(3″)-Ib, aac(6′)-Iaa*	*tet*(B)		*blaCMY-2*		IncFIB, IncFII, IncI1-I(Alpha), IncX1
19–4,593	Kentucky	*aph(6)-Id, aph(3″)-Ib, aac(6′)-Iaa*	*tet*(B)		*blaCMY-2*		IncFIB, IncFII, IncI1-I(Alpha), IncX1
19–4,594	Kentucky	*aph(6)-Id, aph(3″)-Ib, aac(6′)-Iaa*	*tet*(B)		*blaCMY-2*		IncFIB, IncFII, IncI1-I(Alpha), IncX1
19–4,384	Saintpaul	*aac(6′)-Iaa*					
19–4,392	Senftenberg	*aph(6)-Id, aph(3″)-Ib, aac(6′)-Iaa, aph(3′)-Ia, ant(3″)-Ia, ant(2″)-Ia, aadA2*	*tet*(A)	*sul1*	*blaTEM-1B*		Col156
19–4,397	Saintpaul	*aac(6′)-Iaa*					
19–4,411	Saintpaul	*aac(6′)-Iaa*					
19–4,412	Saintpaul	*aac(6′)-Iaa*					
19–4,409	Javiana	*aac(6′)-Iaa*					
19–4,417	Poona	*aac(6′)-Iaa*					
19–4,422	subspecies *houtenae*	*aac(6′)-Iaa*					
S25	Agona	*aac(6′)-Iaa_1*				*fosA7_1_*	

### 3.5 Plasmids

Ten out of eighteen *Salmonella* strains contained at least one plasmid replicon (Table 3). A total of 6 different plasmids replicon sequences were found. Two colicin plasmids, ColpVC and Col156 and four different Inc. plasmids [IncFIB (AP001918), IncFII, IncX1 and Incl1-l (Alpha)] (Table 3). All eight Kentucky strains contained three Inc. plasmids (IncFIB, IncFII, and IncX1), three strains contained an additional plasmid (ColpVc) and three separate strains contained an additional Inc. plasmid (IncI1-I). The Col156 plasmid was present in the *S.* Senftenberg strain.

### 3.6 Prophages

All strains contained prophages, however many of those were incomplete prophages. In six strains we could not detect any intact prophage region. The remaining 12 strains had at least one complete prophage. Three had an additional prophage region (Table 4). Three of the 4 *S.* Kentucky strains contain the Escher_Lys12581Vzw prophage. The other *S.* Kentucky strain contains the prophage Escher_ArgO145. All the *S.* Saintpaul strains and the Javiana strain had the Gifsy-2 prophage. The *S.* Agona and *S.* Senftenberg strain contained the Salmon_SEN8 prophage.

**Table 4:** Number of prophage regions and complete prophages detected in the *Salmonella* strains.

Sample ID	Serotype	Prophage completeness quality			Complete prophage identified	GC content (%)
		# of Questionable prophage regions	# of Incomplete prophage regions	# of Intact prophage regions		
14A	Kentucky	3	5	1	Escher_ArgO145_NC_049918	49.19
14B	Kentucky	3	5	1	Escher_Lys12581Vzw_NC_049917	49.19
16A	Kentucky	3	5	1	Escher_Lys12581Vzw_NC_049917	49.19
16B	Kentucky	3	5	1	Escher_Lys12581Vzw_NC_049917	49.19
19–4,407	Kentucky	2	5	0		
19–4,416	Kentucky	2	4	0		
19–4,426	Kentucky	1	6	0		
19–4,593	Kentucky	2	5	0		
19–4,594	Kentucky	1	6	0		
19–4,384	Saintpaul	2	4	1	Gifsy_2_NC_010393	51.07
19–4,397	Saintpaul	2	5	1	Gifsy_2_NC_010393	51.11
19–4,411	Saintpaul	2	5	1	Gifsy_2_NC_010393	51.11
19–4,412	Saintpaul	2	5	1	Gifsy_2_NC_010393	51.11
19–4,392	Senftenberg	3	1	2	(1) Salmon_SEN8_NC_047753 (2) Salmon_SPN3UB_NC_019545	(1) 49.03 (2) 49.97
19–4,409	Javiana	3	2	2	(1) Gifsy_2_NC_010393 (2) Salmon_SP_004_NC_021774	(1) 51.71 (2) 52.23
19–4,417	Poona	1	3	0		
19–4,422	IV 6,7z4,z24	5	2	2	(1) Salmon_118970_sal3_NC_031940 (2) Salmon_SP_004_NC_021774	(1) 50.24 (2) 53.13
S25	Agona	0	6	1	Salmon_SEN8_NC_047753	49.92

### 3.7 Virulence gene content

All strains contained the highly conserved *Salmonella* Pathogencity Islands (SPI’s) 1–3, with the exception of subspecies *houtenae* strain, which contained only SP1 (Table 5). The subspecies *houtenae* strain was the only strain to contain virulence genes *SpiC* and *SpvB* (Tables 6, 7). All strains with the exception of the 4 *S*. Saintpaul strains, contained Pathogenicity Island C63PI, an iron uptake system. All strains also contained the magnesium uptake system. Fimbrial adhesion genes varied slightly among serotypes. Only the *S*. Javiana strain contained six adhesion genes, whereas subspecies *houtenae* strain contained only two of those adhesion genes.

**Table 5:** *Salmonella* Pathogenicity Islands (SPI’s) present among the *Salmonella* serotypes isolated.

	Salmonella Pathogenicity Islands (SPI’s) present											
Serotype (n)	1	2	3	4	5	8	9	13	14	CS54	C63PI	*PPI (ssaD)
Kentucky (*n* = 9)	9	9	9	9	9	9	9	0	0	0	9	9
Saintpaul (*n* = 4)	4	4	4	4	4	0	4	4	4	4	0	4
Javiana (*n* = 1)	1	1	1	1	1	0	1	1	1	0	1	1
Senftenberg (*n* = 1)	1	1	1	1	1	1	1	0	0	0	1	1
Poona (*n* = 1)	1	1	1	1	1	0	1	1	1	0	1	1
Agona (*n* = 1)	1	1	1	0	1	1	1	0	0	0	1	1
subspecies *houtenae* (*n* = 1)	1	0	0	0	1	0	1	0	1	0	1	0
Total count	18	17	17	16	18	11	18	6	7	4	14	17

*PPI(ssaD), Putative Pathogenicity Island protein (ssaD) gene.

**Table 6:** Virulence genes associated with host invasion and intercellular survival via *Salmonella* type three secretion systems (T3SS’s), among the *Salmonella* serotypes.

	SPI Island 1-T3SS1						SPI Island 2- T3SS2				
Serotypes (n)	*sopA*	*sopB*	*sipA*	*SopE*	*SopE2*	*avrA*	*sifA*	*SpiC*	*SseI*	*SseF*	*PipB*
Kentucky (*n* = 9)	9	9	9	0	7	9	9	0	0	9	9
Saintpaul (*n* = 4)	4	4	4	3	4	4	4	0	0	4	4
Javiana (*n* = 1)	1	1	1	0	1	1	1	0	0	1	1
Senftenberg (*n* = 1)	1	1	1	0	1	1	1	0	0	1	1
Poona (*n* = 1)	1	1	1	0	1	1	1	0	0	1	1
Agona (*n* = 1)	1	1	1	0	1	1	1	0	0	1	1
subspecies *houtenae* (*n* = 1)	0	1	1	1	1	0	1	1	0	1	0
Total count	17	18	18	4	16	17	18	1	0	18	17

**Table 7:** Non-T3SS’s encoded virulence genes among the *Salmonella* serotypes.

	Adhesion Genes						Intracellular survival mechanisms		
	Plasmid-encoded		Fimbrial adherence		Non-fimbrial		Toxin	Serum-resistance	Stress survival
Serotypes (*n*)	*pefA*	*pefB*	*fimA*	*csgA*	*ShdA*	*ratB*	*SpvB*	*rck*	*SodCI*
Kentucky (*n* = 9)	0	4	9	9	9	0	0	0	0
Saintpaul (*n* = 4)	0	4	4	4	4	4	0	0	4
Javiana (*n* = 1)	1	1	1	1	1	1	0	0	0
Senftenberg (*n* = 1)	0	0	1	1	0	0	0	0	0
Poona (*n* = 1)	0	1	1	1	1	1	0	0	0
Agona (*n* = 1)	0	0	1	1	1	0	0	0	0
subspecies *houtenae* (*n* = 1)	0	0	1	1	1	0	1	0	1
Total count	1	10	18	18	17	6	1	0	5

## 4 Discussion

This study is the first in the Cayman Islands, and only the second one in the Caribbean, to utilize Whole Genome Sequencing to determine the epidemiology of *Salmonella* from poultry sources (57). We could isolate *Salmonella* from broilers, layers, imported 1 day old chicks and feral chickens. While the prevalence differed between sample types, overall these differences in confidence intervals were statically insignificant when computed with chi square test. The different serotypes identified in each sample type were isolated with the exception of *S*. Kentucky present in two sample types and the largest diversity in serotypes was seen in samples from layer farms. Samples from bedding/transport material contained only *S.* Kentucky. In free roaming chickens, the dominant serotype was *S*. Saintpaul. This indicates that there is little exchange of strains between the sampled groups. *S*. Kentucky is commonly associated with poultry in North America (11, 18) and the ST152 is dominant sequence type (58, 59). *S.* Saintpaul strains were all ST50, an uncommon strain found in poultry, although it is frequently associated with outbreaks of foodborne infections in the United States (60–62). Our strains however formed a separate group of strains within the ST50, indicating a different epidemiology.

Interestingly, we identified a single *S. houtenae* strain among our feral poultry. This subspecies is commonly associated with cold-blooded animals, but it has also been found in few other animal species (63) and humans (64). It is likely that the strain originated from cold blooded animals, which are common on the Cayman Islands. This is further substantiated by the fact that the other serotypes identified in feral chickens had previously been discovered in the endemic blue iguanas of Grand Cayman (23). Feral chickens thus likely share their *Salmonella* with cold blooded animals on the Cayman Islands.

The most frequently identified serotypes from human salmonellosis in the Caribbean were *S. typhimurium* and *S.* Enteriditis, similar to other parts of the world (65). However, in our samples, we did not detect these serotypes, indicating a low risk of Cayman reared poultry for human health. The isolated *S.* Kentucky strains were clonally related to the North American strains, which is not surprising as U.S hatcheries replenish poultry flocks in the Cayman Islands, under the Cayman Islands Department of Agriculture’s (DOA) permission and regulation. This practice is not unique to the Cayman Islands, as it has been reported that approximately 91% of 27 Caribbean nations, import egg or egg products from a foreign source (66) and recent findings from Trinidad and Tobago also conclude close clustering of *S*. Kentucky strains to strains from a U.S hatchery (67).

All strains had at least the *aac(6′)-Iaa* gene, which is endogenous to the *Salmonella* genus (68). Five strains were MDR. Of those five strains, four were *S*. Kentucky strains and one from the single *S*. Senftenberg strain. These strains contained at least one resistance gene against aminoglycosides, tetracyclines and beta-lactams. The *S.* Senftenberg strain contained an additional resistance gene to sulfonamides. These findings are common in *Salmonell*a (69–71). The predominance of MDR was mainly observed in the day-old chicks imported from North America, a region where MDR is commonly identified in *S.* Kentucky strains (11, 72). More concerning is that in those strains, beta-lactam resistance was mediated by the *bla*_CMY-2_, a plasmid mediated AmpC beta-lactamase which is of clinical importance as they infer resistance to third-generation cephalosporins (73). Plasmids on which this gene has been found are IncA/C and IncI1 plasmids (74). Although we could not locate the gene on a plasmid in our sequences, we suppose it was present on the IncI1 plasmid in our strains, as this plasmid was specifically present in all strains carrying *bla*_CMY-2_.

Striking is also that *Salmonella* from feral chickens had no other AMR other than the *aac(6′)-Iaa* gene, which is present in all *Salmonella* (68), indicating there is no selection for resistance in this population, nor transfer of resistance from other sources. Fosfomycin resistance is rare and was first identified in Canada in *S.* Heidelberg from broilers (75) and is limited to a few other serotypes: *S*. Agona, *S*. Montevideo and *S*. Tennessee (75). This aligns with our findings, as the *fosA7* gene was only present in the single *S.* Agona strain. The *fosA7* gene has been identified outside of Canada, namely the United States (76), China (77), Nigeria (78) and Brazil (79). However, this is the first known reporting of Fosfomycin resistance in *Salmonella* from Caribbean poultry.

Apart from a single *S.* Senftenberg strain, plasmid replicons were only present in the *S*. Kentucky. The plasmids identified in our *S*. Kentucky strains are commonly found in North America and in general carry AMR genes (59, 72, 80, 81), which represents a risk for introducing plasmid carrying resistance genes. This indicates that few plasmids are present in *Salmonella* from feral chickens on the Cayman Islands. This explains also the lack of acquired resistance in these strains as many resistance genes are located on plasmids.

We found that our distribution of serotypes mostly differed from other parts of the world such as China (82, 83), but had similar AMR and virulence genes, particularly with our single *S*. Senftenberg strain (82) and our dominant serotype *S*. Kentucky strains (82, 83). Both serotypes exhibited MDR and AMR genes to beta-lactam antibiotics similarly to ours, but differed in the origin of those AMR genes; with the absence of harboring plasmid replicons in poultry found in China.

However, in these comparisons with our *S*. Kentucky ST152 strains isolated from the Cayman Islands, they differ in their sequence type to the *S*. Kentucky ST198 strains isolated from China. While they may be phenotypically similar in identified AMR and virulence genes, these two sequence types been demonstrated to form genetically distinct lineages (59, 84); ST198 is an international strain and ST152 is one primarily found in North America (84).

The majority of the strains contained at least one intact/functional prophage. Most of the prophage sequences were however not complete. This might be due to the sequencing and assembly. Typically, *S. enterica* subspecies *enterica* serovars contain on average 5 ± 3 prophage regions per genome (85). Gifsy-2 is a common prophage among *Salmonella* serotypes (85) and was represented in all the *S.* Saintpaul and *S.* Javiana strains. Gifsy prophages have been identified to encode virulence gene *SopE* (86), which allows bacterial entry into epithelial cells (87) thus making them more virulent. This virulence gene was present in 3 of the 4 *S*. Saintpaul strains. The other phages, apart from Phage Sen8, found in *S.* Senftenberg, do not contain any known *Salmonella* virulence genes (88).Virulence genes, *Mig-5* and *rck*, have been reported to be typically encoded on IncF plasmids; particularly IncFII (present in our *S*. Kentucky strains), but these genes were absent in our strains. Other major plasmid-encoded virulence genes also identified in IncFII plasmids such as: spv operon (*spvABCDR*) and pef operon (*pefABCD*) (80, 81), were absent in our strains. Only virulence gene, *pefB*, was present in four of the *S*. Kentucky strains.

All strains contained SPIs although they were composed differently. The SPIs and other virulence genes identified do not contribute greatly to the virulence of the strains in relation to infecting humans, as none of the strains are major pathogens for humans.

Due to the low incidence of human salmonellosis on the Cayman Islands, we are limited in assessing the public health risk of our findings. Only 24 cases were reported during 2010–2020. The two most frequently reported NTS serotypes from humans in the Cayman Islands were *S*. Saintpaul (14 cases) and *S*. Poona (5 cases) according to the Health Services Authority^1^. These serotypes were also present in the feral chickens, indicating that these may be a source of human infection together with iguanas. *S*. Saintpaul ST50 has been identified more commonly in clinical infections in humans. There were 2,616 *S*. Saintpaul ST50 strains in Enterobase (89) (*accessed June 25^th^, 2023*), 1,008 from human infections, whereas 176 strains were found in poultry, indicating a potential risk of those strains for human health.

The *S.* Kentucky found in our study is also reported in the United States, although infections with this serotype and the specific ST152 strains are rather rare (90). Probably this serotype does not pose a large public health burden. However, the AMR found in these strains is rather worrisome and are we limited in determining if these AMR genes and plasmids are actively transferred to the local poultry. This could be remedied with a longitudinal study of the life cycle of imported day-old chicks to adulthood, to determine if AMR dissemination occurs. Additionally, we could identify if the serotypes present in the local poultry may also be imported. This research showcases the need for strengthening local policies in food safety and collaborative efforts with all necessary stakeholders to prevent potential foodborne pathogens entering the food supply chain and the burden of illness to public health as envisioned by the One Health concept.

## 5 Conclusion

In conclusion, our study demonstrates that *Salmonella* in broilers and layers on the Cayman Islands are often introduced via the importation of day-old chicks, although other strains also contribute to the prevalence. The presence of *Salmonella* in feral chickens poses a potential risk, given that their serotypes are the ones most commonly isolated from humans in the Cayman Islands. However, due to the strains’ low virulence and the absence of antimicrobial resistance (AMR) and plasmids, the overall health risk may be relatively small. Resistance against antibiotics including *bla*_CMY-2_ gene, is of public health concern. This may warrant stricter import control measures via One Health ideology and also highlights the importance of *Salmonella* surveillance of imported 1 day old chicks and farmed poultry.

## Data availability statement

The original contributions presented in the study are publicly available. This data can be found at: https://www.ncbi.nlm.nih.gov/bioproject/; PRJNA765319.

## Ethics statement

Ethical approval was not required for the study involving animals in accordance with the local legislation and institutional requirements because the study involved fecal droppings from commercial animals and the samples from feral chickens were obtained from culled animals. These animals were culled by the Cayman Island Government instances (Ministry of Health) and we had no other relation to that apart from sampling from the already culled animals.

## Author contributions

SW: Investigation, Writing – original draft. FT: Supervision, Writing – review & editing. HL: Supervision, Writing – review & editing. AJ: Conceptualization, Formal analysis, Methodology, Supervision, Writing – review & editing. PB: Conceptualization, Data curation, Funding acquisition, Methodology, Project administration, Resources, Supervision, Validation, Writing – review & editing.

## Footnotes

^1^https://www.hsa.ky/
